# Interscapular Pain after Anterior Cervical Discectomy and Fusion: Does Zygapophyseal Joints over Distraction Play a Role?

**DOI:** 10.3390/jcm13102976

**Published:** 2024-05-18

**Authors:** Luca Ricciardi, Daniele Bongetta, Amedeo Piazza, Nicolò Norri, Antonella Mangraviti, Sokol Trungu, Evaristo Belli, Luca Zanin, Giorgio Lofrese

**Affiliations:** 1 UOC di Neurochirurgia, AOU Sant’Andrea, Dipartimento NESMOS, Sapienza University of Rome, 00185 Roma, Italy; amedeo.piazza@uniroma1.it (A.P.); norrinicolo@gmail.com (N.N.); antonella.mangraviti@uniroma1.it (A.M.); sokol.trungu@gmail.com (S.T.); 2 SC Neurochirurgia, Ospedale Fatebenefratelli e Oftalmico, 20121 Milan, Italy; daniele.bongetta@asst-fbf-sacco.it; 3 UO di Chirurgia Maxillo-Facciale, AOU Sant’Andrea, Dipartimento NESMOS, Sapienza University of Rome, 00185 Roma, Italy; evaristo.belli@uniroma1.it; 4 Neurosurgery Unit, Department of Medical and Surgical Specialties, Radiological Sciences and Public Health, University of Brescia, 25121 Brescia, Italy; lucazanin00@gmail.com; 5 UOC di Neurochirurgia, Ospedale Bufalini di Cesena, 47521 Cesena, Italy; giorgio.lofrese@gmail.com

**Keywords:** ACDF, rehabilitation, post-operative pain, complications, prosthesis, cervical spine, disc herniation, myelopathy, cervicalgia

## Abstract

**Introduction:** Anterior cervical discectomy and fusion (ACDF) for cervical disc herniation (CDH) is commonly performed. Specific post-operative complications include dysphagia, dysphonia, cervicalgia, adjacent segment disorder, cage subsidence, and infections. However, interscapular pain is commonly reported by these patients after surgery, although its mechanisms have not been clarified yet. **Methods:** This retrospective series of 31 patients undergoing ACDF for CDH at a single Academic Hospital. Baseline and post-operative clinical, radiological, and surgical data were analyzed. The linear regression analysis was conducted to identify any factor independently influencing the incidence rate of post-operative interscapular pain. **Results:** The mean age was 57.6 ± 10.8 years, and the M:F ratio was 2.1. Pre-operative mean VAS-arm was 7.15 ± 0.81 among the 20 patients reporting brachialgia, and mean VAS-neck was 4.36 ± 1.43 among those 9 patients reporting cervicalgia. At 1 month, interscapular pain was still reported by 8 out of the 17 patients who experienced it post-operatively, and it was recovered in all patients after 2 months. The regression analysis showed that interscapular pain was not directly associated with age (*p* = 0.74), gender (*p* = 0.46), smoking status (*p* = 0.44), diabetes (0.42), pre-operative brachialgia (*p* = 0.21) or cervicalgia (*p* = 0.48), symptoms duration (*p* = 0.13), baseline VAS-arm (*p* = 0.11), VAS-neck (*p* = 0.93), or mJOA (*p* = 0.63) scores, or disc height modification (*p* = 0.90). However, the post-operative increase in the mean zygapophyseal joint rim distance was identified as an independent factor in determining interscapular pain (*p* = 0.02). **Conclusions:** Our study revealed that the onset of interscapular pain following ACDF may be determined by over distraction of the zygapophyseal joint rim. Then, proper sizing of prosthetic implants could reduce this painful complication.

## 1. Introduction

In high-income countries, cervical disc herniation (CDH) and degenerative cervical spondylosis (DCS) represent common clinical conditions determining pain and functional impairment. The prevalence of CDH ranges from 2.5 to 5 cases per 100,000 subjects, and 10–15% of the cases may eventually require surgical management over time. While neurological deficits represent indications for surgery, conservative management is usually the first line of treatment in case of painful conditions providing progressive functional impairment [1,2,3,4,5,6]. 

At the clinical examination, patients may report cervical and brachial pain and sensory dysfunction, consisting of paresthesia or dysesthesia in a single- or multiple-radicular territory, some degree of impairment of hand and finger movements, and restricted neck range of motion. The physician investigates neurological signs of myelopathy or nerve root palsy.

Conservative management consists of physical therapy protocols and medications aiming to reduce the pain rate while restoring functional status. Conventionally, this strategy is pursued for 3 to 6 months before considering surgical interventions [7,8].

In cases of neurological deterioration or failure of conservative treatment, surgery is discussed as an option with patients. During the last two decades, anterior approaches to the cervical spine have been progressively preferred to posterior ones, according to the clinical-radiological outcome and complications rate. Anterior cervical discectomy and fusion (ACDF) is now considered the standard surgical procedure for cervical disc herniation, and it has been progressively supplanting the anterior cervical discectomy (ACD), which was related to higher risks of segmental kyphosis and post-operative cervical pain [1,3,4,5,6,7,9]. 

The primary reported risks related to ACDF are surgical site infections, peri-operative hematoma, inferior laryngeal nerve palsy, dysphagia, esophageal lesions, implant failure, and adjacent segment disorder/disease [3,4,7,10,11,12,13,14,15]. Nonetheless, it is commonly experienced in these patients some degree of post-operative interscapular pain, which usually self-recovers in two months [16]. However, patients frequently complain about this symptom, which is usually not reported pre-operatively. Furthermore, it is poorly discussed, and they might not be informed about this post-operative complication. The prevalence of this phenomenon is not well established in the literature since few papers report it as a complication or consequence after ACDF. Standard clinical scales and questionnaires for cervical spine disorders do not include interscapular pain; then, physicians might systematically underreport it in their clinical practice and scientific papers.

The present investigation aims to identify any risk factors for developing interscapular pain after ACDF for CDH or CDS. We hypothesize that the use of implants higher than needed may determine the grade of zygapophyseal joints over distraction, resulting in post-operative interscapular pain. This painful condition might be determined by the joint capsule distraction and their nociceptive fiber stimulation, which are non-responsive to medications and PhT protocols.

## 2. Materials and Methods

### 2.1. Study Design

The present study is a retrospective investigation conducted at a single academic institution. All patients signed an informed consent form, including the authorization for their anonymous data analysis for scientific purposes. The IRB approved the retrospective data collection with the protocol registration number—Prot. 3195/2024—28.02.2024.

The institutional database was screened for hospitalized patients diagnosed with “cervical spondylosis” and “cervical disc herniation”, “cervicalgia”, and “brachialgia”, and “cervical nerve root palsy” and “cervical myelopathy”, then filtered according to the unique surgical procedure identified for inclusion, which was “anterior cervical discectomy and fusion”.

Patients were considered for eligibility if complete medical–radiological documentation was available on the informatic system. The following inclusion criteria were adopted: age > 18 years, single cervical level disc herniation determining cervical nerve root palsy and cervical myelopathy, and cervical-brachial pain from three months after failing the conservative treatment, clinical follow-up 12 months. Exclusion criteria were a history of previous cervical spine surgery or traumatic injuries prior to the current hospitalization, interscapular pain or ache at the cervical–thoracic junction reported during the pre-operative evaluation, fibromyalgia, oncological disease, or surgical procedure different from single-level ACDF without plating. A time range for screening was set from January 2019 to December 2022. 

### 2.2. Surgical Technique

All the patients included in the present investigation were operated on using the standardized institutional technique. Patients were supine with a gentle neck extension on a C-shaped head holder. Shoulders were fixed to the surgical table using Tensoplast for gently lifting them caudally, without stretching, to avoid brachial plexus injuries. The surgical approach was always performed on the left side, using a 3 to 5 cm horizontal paramedian incision exploiting a neck-skin wrinkle projecting on the target disc space, as intra-operatively verified with fluoroscopy [17]. A standard Smith–Robinson approach was used for reaching the anterior cervical spine, and a Caspar self-retaining soft-tissue retractor was placed with its tips underneath the medial aspects of the anterior longus colli muscles, which were adequately detached from their insertion. Somatic pins (14 mm) were placed and gently retracted using the Caspar self-retaining somatic retractor, avoiding any over-distraction through a fluoroscopy check. Discectomy was performed under exoscopic magnification (B-Braun Aesculap, Melsungen, Germany) using dedicated micro-instruments such as rongeur, curette, and forceps while avoiding the use of the high-speed drill to minimize the risk of violating and injuring the endplates. The posterior longitudinal ligament was sectioned in all the cases. A probe was used for implant sizing to identify the minimum prosthesis height capable of resisting the surgeon’s gentle pulling after releasing the inter somatic Caspar retraction. A stand-alone carbon implant (Brantigan—Depuy, Raynham, MA, USA) cage filled with a synthetic, bioactive, and osteoconductive bone void filler (Attrax Putty—Nuvasive, San Diego, CA, USA) was implanted under fluoroscopic guidance [18,19,20]. Subfascial drainage was always placed and removed on the first post-operative day. Cervical orthosis was never prescribed [2,21].

### 2.3. Clinical and Radiological Outcome

From our Department of Neurosurgery and Spine Surgery dataset, we anonymously retrieved the demographics of eligible patients. Symptoms and duration were registered at the admission, along with the ten-point itemized visual analog scale (VAS) score for cervical and brachial pain and the mJOA score. Clinical outcome was evaluated regarding the onset of interscapular pain post-operatively and its duration, peri-operative complications, and through 1, 6, and 12 month mJOA scores calculated during outpatient follow-up. 

**Figure 1:**
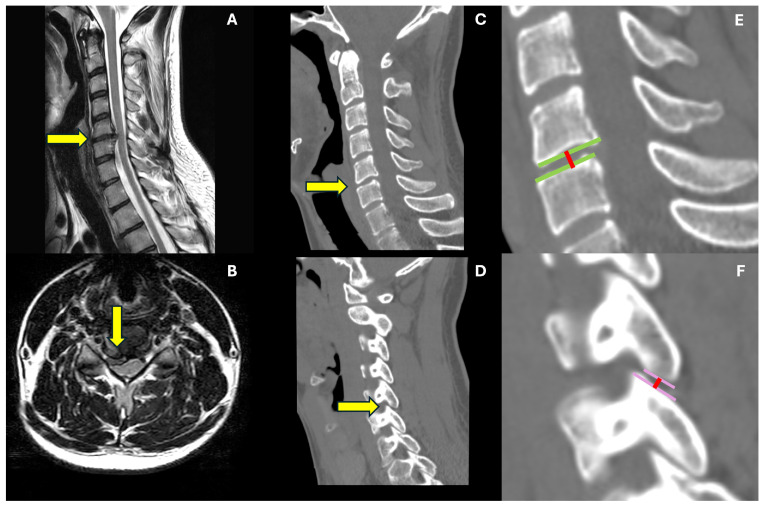
Case illustration—radiological measurements. This is an illustrative case of a 32-year-old male suffering from C5-6 right paramedian cervical disc herniation, determining C7 radicular pain and triceps palsy. In (**A**,**B**), sagittal and axial t2-weighted 1.5 T MRI, respectively, we see identified by the yellow arrow the herniated disc and no significant degenerative condition of the rest of the cervical spine. In (**C**,**D**), we find the CT scan exam, including sagittal median (**C**) and lateral (**D**) cuts. The (**E**) is a zoomed image of the C5-6 level, where we illustrate how we measure di discal space height (red line) at the mean point of the opposite endplates (green lines). The (**F**) is a zoomed visualization of the zygapophyseal joint; in this the joint rims are identified by the pink lines, and the distance is measured at their mean point (red line).

All patients were evaluated through a pre-operative cervical spine C.T. scan and MRI at admission. In contrast, a 1 month C.T. scan and cervical spine x-rays 6 and 12 months after surgery were required to assess radiological outcomes. Level of disc herniation was identified on the pre-operative MRI, while the disc height, defined as the distance between the upper and lower endplate measured at their middle point, the segmental zygapophyseal joint (ZAJ) distance, considered as the gap between the cortical rims of the joint measured at their middle point (mean measurement between the R and L distance), and global cervical lordosis were calculated on pre- and post-operative C.T. scans and x-rays, respectively. Cervical fusion was considered to have a Cobb angle variation < 4° and an interspinous process distance variation < 2 mm in dynamic x-rays or bone bridges on lateral view x-rays [22]. One single experienced spine surgeon (L.R.) performed all the measurements on the institutional picture archiving and communication system (PACS). Measurements are illustrated in Figure 1.

### 2.4. Statistics and Data Analysis

Values were reported as the mean ± standard deviation. The student *t*-test was used to compare the quantitative continuous variables. Fisher’s exact test (2-sided) was used to compare categorical variables. Cohen’s d and size effect were measured. Statistical significance was predetermined at an alpha level of 0.05. Univariate and multivariate multiple regression analyses were performed. StatPlus (AnalystSoft Inc., Brandon, FL, USA) was used for data analysis. The standard error of measurement (SEM) and intra-class correlation coefficient (ICC) for ZAJ distance were calculated on the repeated measurement (two measurements in two consecutive days in a random manner). The measurements were performed by a single board-certified spine surgeon with more than ten years of experience in spine surgery.

## 3. Results

**Figure 2:**
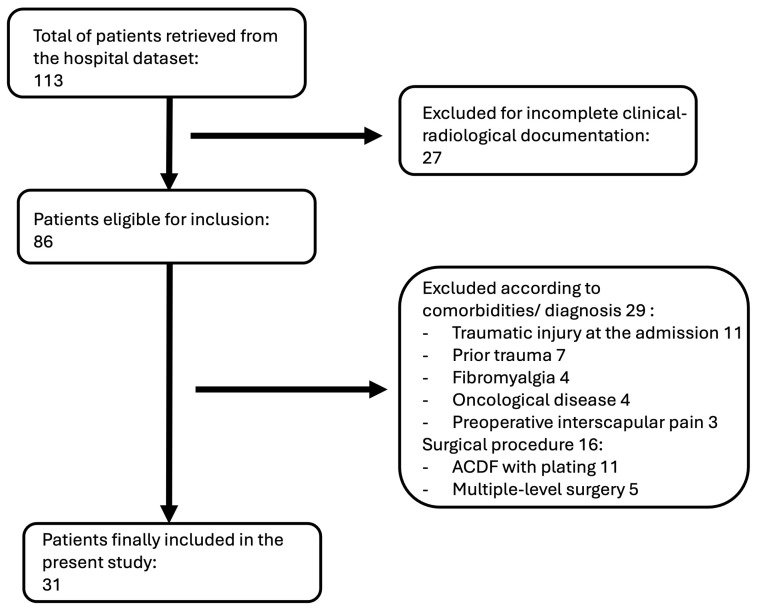
Patients selection.

The institutional dataset was screened according to the selection criteria in Section 2. A total of 113 patients were eligible for the present study; 72 were excluded according to the inclusion criteria (29 diagnoses, 27 incomplete clinical–radiological documentation, and 16 surgical procedures). Patients’ selection and exclusions with reasons are resumed in Figure 2. A total of 31 patients were included in the present investigation. The mean age was 57.6 ± 10.8 years, the M:F ratio was 2.1 (21 M:10 F), 7 out of the 31 patients were smokers (22.6%), and diabetes was reported in 5 patients (16.1%). 

Brachialgia was reported by 20 patients (64.5%), cervicalgia by 9 (29%), and cervical-brachialgia by 6 (19.4%). The pre-operative mean VAS-arm was 7.15 ± 0.81 among the 20 patients reporting brachialgia, and the mean VAS-neck was 4.36 ± 1.43 among those nine patients reporting cervicalgia. Cervical myelopathy was diagnosed in 7 patients (22.6%), with three suffering from brachialgia (9.68%) and four suffering from cervicalgia (12.9%). 

The mean pre-operative duration of symptoms (cervicalgia and/or brachialgia) was 7.04 ± 2.68 months, while the mean mJOA score at admission among the seven patients with myelopathy was 14.29 ± 1.60—see Table 1.

### 3.1. Surgical Data

The operated cervical levels were C3-4 in 4 (12.9%) patients, C4-5 in 12 (38.7%) patients, C5-6 in 10 (32.3%) patients, and C6-7 in 5 (16.1%) patients. Implant height was 5 mm in 15 patients, 6 mm in 14, and 7 mm in 2. The standard width/depth size (15 × 12 mm) was used in all the cases. No intra-operative complications were recorded.

### 3.2. Clinical Outcome

In one patient, the intradermic suture failed in 2 days, and re-suturing was needed. Five patients reported post-operative pain at the surgical site, but it self-recovered in 24 h, while eight patients complained of dysphagia, although not limiting solid or liquid deglutition and self-recovering within seven days in all of them. There were no cases of post-operative dysphonia, surgical site infection, or implant failure in this series. Interscapular pain arose in 17 cases (54.8%). In 4 out of the 17 patients, this symptom developed within 24 h from the surgical procedure, while in the other 13, it was reported on the second or third post-operative day. One month after surgery, the mean VAS-arm score among patients with pre-operative brachialgia was 2.7 ± 0.92, the mean VAS-neck score among those with cervicalgia was 1 ± 1.10, and interscapular pain was still present in 8 out of the 17 patients who experienced it post-operatively. 

Six months after surgery, the mean VAS-arm score among patients with pre-operative brachialgia was 0.25 ± 0.44 (Cohen’s d: 3.4, effect-size: 0.86), the mean VAS-neck score among those with pre-operative cervicalgia was 0.27 ± 0.9 (Cohen’s d: 0.73, effect-size: 0.34), and the mean mJOA score among myelopathic patients was 15 ± 1.73 (Cohen’s d: −0.42, effect-size: −0.21). Any patient no longer reported post-operative interscapular pain, and those who suffered from it declared that it self-recovered within two months after surgery.

At the twelve-month follow-up, the mean VAS-arm score among patients pre-operatively suffering from brachialgia was 0.1 ± 0.45 (Cohen’s d: 10.76, effect-size: 0.98), while the mean VAS-neck among those complaining of cervicalgia at the admission was 0.27 ± 0.91 (Cohen’s d: 3.41, effect-size: 0.86). The mean mJOA score among myelopathic patients was 15.43 ± 0.72 (Cohen’s d: −0.91, effect-size: −0.42)—see Table 2.

### 3.3. Radiological Outcome

The mean cervical lordosis was 24.13° ± 3.37° pre-operatively and 24.65° ± 2.52° post-operatively, with no significant differences between pre- and post-operative values (*p* = 0.50) (Cohen’s d: −0.18, effect-size: −0.09). 

The mean disc space height at the disc herniation level was 5.19 mm ± 0.32 in those patients who had experienced post-operative interscapular pain and 5.02 mm ± 0.43 in those who did not. No significant differences existed between the two subgroups regarding pre-operative disc height (*p* = 0.22) (Cohen’s d: 0.45, effect size: 0.22).

After surgery, the mean disc height at the operated level was 5.46 mm ± 0.48, with a significant increase compared to pre-operative measurement (*p* < 0.01) (Cohen’s d: −0.81, effect-size: −0.38). The subgroup analysis showed that the mean post-operative disc height in patients reporting interscapular pain was 5.71 mm ± 0.44 (Cohen’s d: −1.37, effect-size: −0.57), showing a significant improvement compared to their mean pre-operative height (*p* < 0.01). A mean height of 5.15 mm ± 0.32 was measured among those patients not reporting interscapular pain, showing no significant differences compared to pre-operative measurements (*p* = 0.49) (Cohen’s d: −0.35, effect-size: −0.17). 

At the admission, the mean ZAJ distance at the disc herniation level was 1.05 mm ± 0.14 in those patients who had experienced post-operative interscapular pain and 0.99 mm ± 0.13 in those who did not. No significant differences existed between the two subgroups regarding pre-operative ZAJ mean distance (*p* = 0.23) (Cohen’s d: 0.44, effect-size: 0.22).

The mean post-operative ZAJ distance was 1.22 mm ± 0.20, with a significant increase compared to the pre-operative measurement (*p* < 0.01) (Cohen’s d: −1.18, effect-size: −0.51). The subgroup analysis showed that the mean post-operative ZAJ distance in patients reporting interscapular pain was 1.36 mm ± 0.14, showing a significant improvement compared to their mean pre-operative height (*p* < 0.01) (Cohen’s d: −2.21, effect-size: −0.74), and a mean height of 1.05 mm ± 0.11 among those who did not report interscapular pain, showing any significant differences when compared to pre-operative measurements (*p* = 0.20) (Cohen’s d: −0.5, effect-size: −0.24). See Table 3.

The standard error of measurement (SEM) and the intra-class correlation coefficient (ICC) for ZAJ distance were 0.1 mm and 0.86, respectively. The SEM is more than ten times lower than the observed difference between the two groups, and the ICC confirms the “good reliability” of the measurements.

No mobilizations of the implants were detected at follow-up, while fusion of the treated level was appreciated in all the patients at the 12 month x-rays—see Table 4.

## 4. Discussion

The present retrospective study confirmed our hypothesis that the use of higher implants in the discal space during ACDF determines posterior over-distraction of ZAJ, which underlay the post-operative appearance of interscapular pain. The multivariate analysis confirmed that ZAJ modification represents the only independent factor influencing the onset of post-operative interscapular pain.

Cervical disc herniation represents a common cause of disability due to painful conditions and/or neurological impairment, eventually affecting functional status. High-income countries are burdened by CDH direct and indirect costs related to disability, specialistic evaluations, diagnostics, hospitalization, surgeries, rehabilitation, and sick leaves. Therefore, there is a high medical and social interest in identifying any factor potentially influencing clinical outcomes [22].

Cervical nerve root palsy, cervical and/or arm pain resistant to conservative strategies, and cervical myelopathy are generally accepted as indications for surgery in cases of cervical disc herniation [7]. 

Other than surgical-related peri- and post-operative complications, patients are particularly demanding to manage, especially for post-operative pain rate and early functional recovery, despite the current minimally invasive techniques and medical-anesthesiologic advancements. Accordingly, physicians are also required to minimize post-operative discomfort. Therefore, identifying factors potentially affecting patients’ postsurgical comfort, satisfaction, and early functional restoration should be identified and conceived as part of the treatment itself [9,23].

It is commonly experienced by spine surgeons that post-operative interscapular pain may occur in patients undergoing ACDF procedures. This localized ache usually occurs within the first 48–72 h after surgery, and it may negatively influence the early outcome, forcing bed rest and discouraging early and complete mobilization [16]. This painful condition is usually poorly responsive to common medications, such as NSAIDs and painkillers, while it normally self-recovers within two months after surgery. Although patients often fully recover from pre-operative symptoms, they complain about the interscapular pain that was not present at admission and may eventually counteract the post-operative overall amelioration. 

The present retrospective investigation aimed to identify any potential factors influencing the onset of interscapular pain after elective ACDF for CDH. Accordingly, the retrospective study was designed to retrieve demographical, clinical, and radiological data of patients scheduled for elective ACDF surgery in a time range of 3 years at a single Academic Hospital. The minimum follow-up for inclusion was set at one year, according to the characteristics of the assessed condition. 

The primary outcome of this study was to evaluate the incidence of post-operative interscapular pain and any possible factors independently increasing the chances of experiencing it. 

Postsurgical interscapular pain was experienced and reported by 17 (54.8%) of the 31 included patients. In all cases, it appeared in the first three days after surgery: 4 cases in 24 h and 14 cases in the subsequent 48 h. All the patients reported a spontaneous resolution of the interscapular discomfort within one month (9 patients) or two months (8 patients). 

The analysis of demographical data showed that neither age, gender, smoking status, nor diabetes influenced the onset of post-operative interscapular pain. Nonetheless, baseline clinical data, such as type of pain (cervicalgia and/or brachialgia) and its intensity (VAS-neck and VAS-arm), symptoms duration, and severity of myelopathy, were not identified as factors influencing interscapular pain appearance after surgery. 

**Figure 3:**
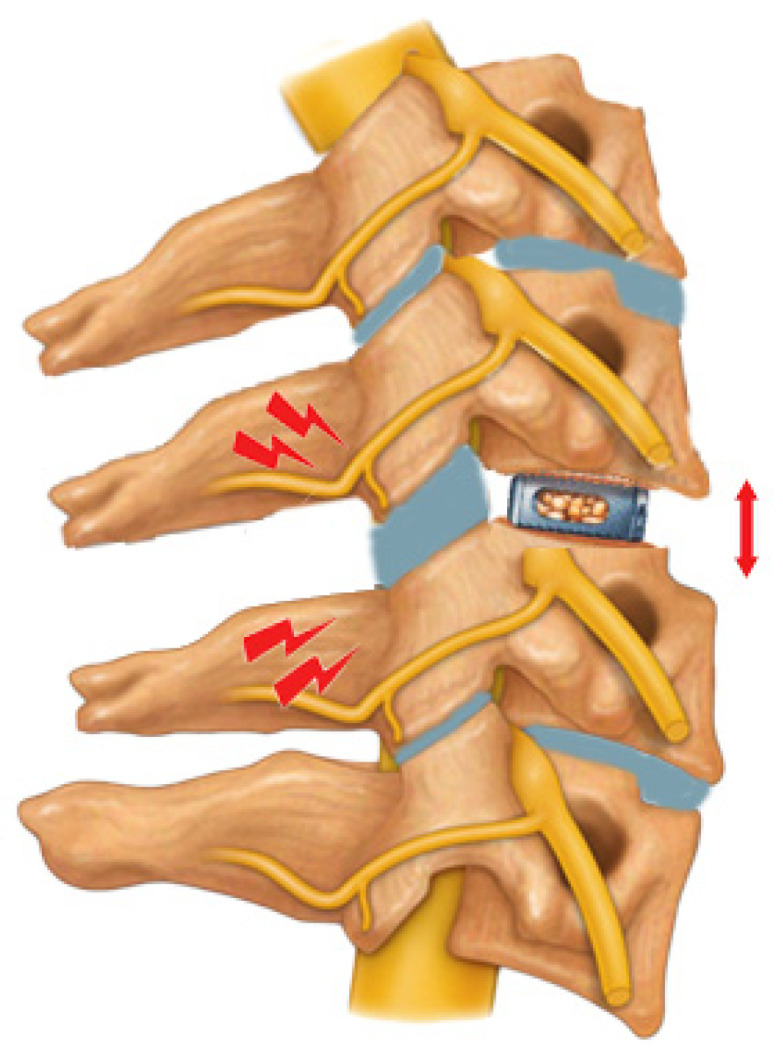
Zygapophyseal joints over distraction after ACDF and post-operative interscapular pain. In this drafting, the cage is implanted at the herniated disc level, according to the ACDF technique. The red arrow highlights how the oversized implant increases the intervertebral space height compared to contiguous ones, and this determines some degree of posterior over-distraction of the segmental zygapophyseal joints and their capsule, which may underlie the post-operative interscapular pain occurrence.

Contrarywise, going through the radiological data, while the pre- to post-operative variation in the disc space height at the operated level and cervical lordosis variation were not independently associated with the post-operative interscapular pain, our study revealed how the over-distraction of the segmental zygapophyseal joints at the operated level may represent a higher risk for developing post-operative interscapular pain—See Figure 3. 

The implant of a cage (red arrow), which determines the posterior over-distraction of the homo-segmental zygapophyseal joints (red bolts), may underlie the pathogenesis of the post-operative interscapular pain, as we found in the multivariate analysis of the raw data from this case series.

According to the linear regression analysis, the pre-to-post-operative cervical lordosis modification was not statistically significant and was not associated with post-operative interscapular pain.

The critical analysis of these results seems to suggest that a single factor may play a major role in this phenomenon, while we cannot exclude that any other factor not evaluated in the present study may participate in this. However, zygapophyseal joint distraction grade is not standardly evaluated after ACDF; this could have been under-noticed in previous investigations.

The rationale of our results can be easily identified in the capsular over-distraction of these synovial joints, in which the nociceptive innervation of their capsules could underlie the clinical phenomenon of interscapular pain after ACDF. The etiopathogenesis of such a condition could be related to the choice of cages higher than the original inter somatic space or determined by some grade of posterior distraction when operating on the anterior column. The self-recovering scenario in a few weeks could be explained as the progressive spontaneous accommodation of the treated segment and a sort of compensation by the adjacent cervical levels. Nonetheless, identifying the mechanism underlying the post-operative interscapular pain may influence the rehabilitation protocols, leading to faster symptom recovery [12,24]. Furthermore, reducing the incidence of this post-operative painful condition may reduce the costs related to medications and physiotherapy, which also seem to be ineffective in terms of pain severity and duration.

Our radiological data suggest that a few millimeters of over-distraction may lead to post-operative interscapular pain, and this could not be detected using intra-operative fluoroscopy. Nevertheless, even when using an intra-operative C.T., the detection of a few millimeters over the distraction of the posterior ZAJ would be difficult, and it should be carefully considered for evaluating implant substitution. Furthermore, the use of cages higher than the estimated original disc height has not been reported to ameliorate clinical-radiological outcomes. Therefore, prosthetic implants fitting the space without providing segmental over-distraction, followed by a posterior stretching of the zygapophyseal joints, should be preferred in these cases. 

The standard use of intra-operative C.T. scans for verifying implant size and ZAJ distraction grade may reduce the risk for post-operative interscapular pain once the underlying mechanism we are hypothesizing in the present paper is verified in future studies [25,26]. Lastly, further studies with longer follow-ups are needed to better investigate the effect of segmental lordosis over-correction and over-distraction on global cervical alignment and biomechanics.

### Limitations

There are limitations to the present investigation that should be carefully considered for a proper interpretation of its results. Firstly, this is a retrospective study, and its design influences its quality and level of evidence; secondly, our results are based on a relatively small patient sample from a single institution, eventually limiting the power of the analysis; thirdly, the primary outcome (post-operative interscapular pain) was retrieved from the clinical documentation and outpatient follow-up; while it is not part of any standard form, we should consider that some patients may experience it without reporting to the physician and could not have been specifically asked for it; fourthly, segmental lordosis was not included in the analysis, and this was already reported as a relevant factor influencing the clinical and radiological outcome. Fifthly, smoking frequency and duration, BMI, and education level were not retrievable from the clinical data, and these may represent supplementary variables potentially influencing clinical–radiological outcomes; lastly, there is not a score for interscapular pain, and we were able to retrieve only its presence/absence in a dichotomous manner. Our relatively small sample did not allow us to conduct a subgroup analysis to evaluate the role of demographics and clinical characteristics in affecting outcomes. Accordingly, our results should be considered informative data on a clinical phenomenon following ACDF procedures for CDH. Its impact on patients’ outcomes and influence on clinical practice is not yet established, and higher-quality studies are strictly needed to evaluate this clinical entity and any potential research interest in this topic.

## 5. Conclusions

Post-operative interscapular pain after ACDF is commonly experienced by patients, although this is poorly discussed as a complication while commonly considered a transitory consequence. Nonetheless, this may affect the post-operative surgery-related quality of life, eventually affecting patients’ satisfaction and functional restoration after surgery, regardless of pre-operative symptom regression. Our data suggest that ZAJ distraction due to segmental over-distraction may underlie this symptomatology, which usually recovers within two months. An intra-operative CT scan for properly evaluating implant size and local anatomy modification might prevent this painful consequence. Further, properly designed prospective studies are needed to validate our results.

## Figures and Tables

**Table 1:** Demographics and symptoms.

Patients (n°)	31
Age (mean ± SD)	57.61 ± 10.85
Gender	21 M:10 F
Smoking status	7 (22.6%)
Diabetes	5 (16.1%)
Brachialgia	20 (64.5%)
Cervicalgia	9 (29%)
Brachialgia and Cervicalgia	6 (19.4%)
Symptoms Duration (months)	7.04 ± 2.68

**Table 2:** Clinical data.

	Pre-Op	1 Month	6 Months	12 Months	Cohen’s d	Effect-Size	*p*-Value
VAS-neck	4.36 ± 1.43	1 ± 1.10	0.27 ± 0.9	0.27 ± 0.91	3.41	0.86	<0.01
VAS-arm	7.15 ± 0.81	2.7 ± 0.92	0.25 ± 0.44	0.1 ± 0.45	10.76	0.98	<0.01
mJOA	14.29 ± 1.60	N/A	15 ± 1.73	15.43 ± 0.72	−0.91	−0.42	<0.01

VAS: visual analog scale; mJOA: modified Japanese Orthopedic Association score. *p*-value, Cohen’s d, and effect-size refer to the pre-operative to 12 month follow-up comparison.

**Table 3:** Radiological data.

	Pre-Operative	Post-Operative	Cohen’s d	Effect-Size	*p*-Value
DISC SPACE (mm)	5.11 ± 0.38	5.46 ± 0.48	−0.81	−0.38	<0.01
Interscapular pain +	5.19 ± 0.32	5.71 ± 0.44	−1.37	−0.57	<0.01
Interscapular pain −	5.02 ± 0.43	5.15 ± 0.32	−0.35	−0.17	0.37
Zygapophyseal Joint Distraction (mm)	1.03 ± 1.22	1.22 ± 0.2	−1.18	−0.51	<0.01
Interscapular pain +	1.05 ± 0.14	1.36 ± 0.14	−2.21	−0.74	<0.01
Interscapular pain −	0.99 ± 0.13	1.05 ± 0.11	−0.5	−0.24	0.20
Cervical lordosis (°)	24.13 ± 3.37	24.65 ± 2.52	−0.18	−0.09	0.50

**Table 4:** Multivariate analysis.

	Coeff.	Standard Error	Stat t	Significance
Intercept	0.321582044	1.69588721	0.189624665	0.851848927
Age	−0.00389076	0.008196673	−0.474675516	0.641058821
Gender	0.113253252	0.190619362	0.594132992	0.560247305
Smoking	−0.029197792	0.230930775	−0.126435256	0.900870753
Diabetes	−0.100450733	0.239643276	−0.419167752	0.680342267
Brachialgia	1.72944055	1.062010835	1.628458479	0.121817082
Cervicalgia	−0.027553088	0.236673029	−0.116418368	0.908685221
Duration	0.031543204	0.040760078	0.773874967	0.449633496
VAS-a t0	−0.228626283	0.139998016	−1.633068027	0.120837308
VAS-n t0	0.043997651	0.158370071	0.27781544	0.784504462
mJOA t0	−0.014048917	0.060605166	−0.231810556	0.819452551
CL modification	0.020323289	0.067315898	0.301909202	0.766383498
DH mod	0.023703603	0.243234903	0.097451485	0.923507601
ZAJ mod	1.749252615	0.691751425	2.528730048	* 0.021635343

VAS: visual analogue scale; mJOA: modified Japanese Orthopedic Association score; CL: cervical lordosis; DH: disc height; ZAJ: zygapophyseal joint. * *p*-value < 0.05.

## Data Availability

Data will be provided on request after the approval of the local IRB for data sharing.

## References

[1] Sahai N., Changoor S., Dunn C.J., Sinha K., Hwang K.S., Faloon M., Emami A. (2019). Minimally Invasive Posterior Cervical Foraminotomy as an Alternative to Anterior Cervical Discectomy and Fusion for Unilateral Cervical Radiculopathy: A Systematic Review and Meta-Analysis. Spine.

[2] Ricciardi L., Scerrati A., Olivi A., Sturiale C.L., De Bonis P., Montano N. (2020). The Role of Cervical Collar in Functional Restoration and Fusion after Anterior Cervical Discectomy and Fusion without Plating on Single or Double Levels: A Systematic Review and Meta-Analysis. Eur. Spine J..

[3] Montano N., Ricciardi L., Olivi A. (2019). Comparison of Anterior Cervical Decompression and Fusion versus Laminoplasty in the Treatment of Multilevel Cervical Spondylotic Myelopathy: A Meta-Analysis of Clinical and Radiological Outcomes. World Neurosurg..

[4] Shangguan L., Ning G.-Z., Tang Y., Wang Z., Luo Z.-J., Zhou Y. (2017). Discover Cervical Disc Arthroplasty versus Anterior Cervical Discectomy and Fusion in Symptomatic Cervical Disc Diseases: A Meta-Analysis. PLoS ONE.

[5] Byvaltsev V.A., Stepanov I.A., Riew D.K. (2020). Mid-Term to Long-Term Outcomes After Total Cervical Disk Arthroplasty Compared With Anterior Diskectomy and Fusion: A Systematic Review and Meta-Analysis of Randomized Controlled Trials. Clin. Spine Surg..

[6] Zavras A.G., Federico V.P., Butler A.J., Nolte M.T., Dandu N., Phillips F.M., Colman M.W. (2024). Relative Efficacy of Cervical Total Disc Arthroplasty Devices and Anterior Cervical Discectomy and Fusion for Cervical Pathology: A Network Meta-Analysis. Global Spine J..

[7] Feng S., Zheng B., Zhang L., Wang W. (2021). A Systematic Review and Meta-Analysis Compare Surgical Treatment and Conservative Treatment in Patients with Cervical Spondylotic Myelopathy. Ann. Palliat. Med..

[8] Atalay B., Gadjradj P.S., Sommer F.S., Wright D., Rawanduzy C., Ghogawala Z., Härtl R. (2023). Natural History of Degenerative Spondylolisthesis: A Systematic Review and Meta-Analysis. World Neurosurg..

[9] Goh G.S., Yue W.-M., Guo C.-M., Tan S.-B., Chen J.L.-T. (2021). Does the Predominant Pain Location Influence Functional Outcomes, Satisfaction and Return to Work After Anterior Cervical Discectomy and Fusion for Cervical Radiculopathy?. Spine.

[10] Quinto E.S., Paisner N.D., Huish E.G., Senegor M. (2024). Ten-Year Outcomes of Cervical Disc Arthroplasty Versus Anterior Cervical Discectomy and Fusion: A Systematic Review With Meta-Analysis. Spine.

[11] Takase H., Haze T., Yamamoto D., Inagaki N., Nitta M., Murata H., Yamamoto T. (2024). Network Meta-Analysis of C5 Palsy After Anterior Cervical Decompression of Three to Six Levels: Comparing Three Different Procedures. Spine.

[12] Moustafa I.M., Diab A.A., Harrison D.E. (2022). The Efficacy of Cervical Lordosis Rehabilitation for Nerve Root Function and Pain in Cervical Spondylotic Radiculopathy: A Randomized Trial with 2-Year Follow-Up. J. Clin. Med..

[13] Jaeger J., Moore R., Smith W., Wu C., Oakley P., Harrison D. (2022). Synergistic treatment methods of structural rehabilitation (cbp®) and neurosurgery maximizing pre- and post-operative cervical lordosis and patient outcome in cervical total disc replacement. J. Contemp. Chiropr..

[14] Ricciardi L., Scerrati A., Bonis P.D., Miscusi M., Trungu S., Visocchi M., Papacci F., Raco A., Proietti L., Pompucci A. (2021). Long-Term Radiologic and Clinical Outcomes after Three-Level Contiguous Anterior Cervical Diskectomy and Fusion without Plating: A Multicentric Retrospective Study. J. Neurol. Surg. Part A Cent. Eur. Neurosurg..

[15] Nijim W., Cowart J.H., Banerjee C., Postma G., Paré M. (2023). Evaluation of Outcome Measures for Post-Operative Dysphagia after Anterior Cervical Discectomy and Fusion. Eur. Arch. Oto-Rhino-Laryngol..

[16] Xie J., Hurlbert R.J. (2007). Discectomy versus Discectomy with Fusion versus Discectomy with Fusion and Instrumentation: A Prospective Randomized Study. Neurosurgery.

[17] Miscusi M., Bellitti A., Peschillo S., Polli F.M., Missori P., Delfini R. (2007). Does Recurrent Laryngeal Nerve Anatomy Condition the Choice of the Side for Approaching the Anterior Cervical Spine?. J. Neurosurg. Sci..

[18] Lehr A.M., Oner F.C., Delawi D., Stellato R.K., Hoebink E.A., Kempen D.H.R., van Susante J.L.C., Castelein R.M., Kruyt M.C. (2020). Dutch Clinical Spine Research Group Efficacy of a Standalone Microporous Ceramic Versus Autograft in Instrumented Posterolateral Spinal Fusion: A Multicenter, Randomized, Intrapatient Controlled, Noninferiority Trial. Spine.

[19] Lehr A.M., Oner F.C., Delawi D., Stellato R.K., Hoebink E.A., Kempen D.H.R., van Susante J.L.C., Castelein R.M., Kruyt M.C. (2020). Dutch Clinical Spine Research Group Increasing Fusion Rate Between 1 and 2 Years After Instrumented Posterolateral Spinal Fusion and the Role of Bone Grafting. Spine.

[20] Berjano P., Langella F., Damilano M., Pejrona M., Buric J., Ismael M., Villafañe J.H., Lamartina C. (2015). Fusion Rate Following Extreme Lateral Lumbar Interbody Fusion. Eur. Spine J..

[21] Ricciardi L., Stifano V., D’Arrigo S., Polli F.M., Olivi A., Sturiale C.L. (2019). The Role of Non-Rigid Cervical Collar in Pain Relief and Functional Restoration after Whiplash Injury: A Systematic Review and a Pooled Analysis of Randomized Controlled Trials. Eur. Spine J..

[22] Obermueller T., Wagner A., Kogler L., Joerger A.-K., Lange N., Lehmberg J., Meyer B., Shiban E. (2020). Radiographic Measurements of Cervical Alignment, Fusion and Subsidence after ACDF Surgery and Their Impact on Clinical Outcome. Acta Neurochir..

[23] Nin D.Z., Chen Y.-W., Kim D.H., Niu R., Powers A., Chang D.C., Hwang R.W. (2024). Healthcare Costs Following Anterior Cervical Discectomy and Fusion or Cervical Disc Arthroplasty. Spine.

[24] Parker S.L., Godil S.S., Shau D.N., Mendenhall S.K., McGirt M.J. (2013). Assessment of the Minimum Clinically Important Difference in Pain, Disability, and Quality of Life after Anterior Cervical Discectomy and Fusion: Clinical Article. J. Neurosurg. Spine.

[25] Bębenek A., Dominiak M., Godlewski B. (2023). Cervical Sagittal Balance: Impact on Clinical Outcomes and Subsidence in Anterior Cervical Discectomy and Fusion. Biomedicines.

[26] Battistelli M., Polli F.M., D’Alessandris Q.G., D’Ercole M., Izzo A., Rapisarda A., Montano N. (2023). An Overview of Recent Advances in Anterior Cervical Decompression and Fusion Surgery. Surg. Technol. Int..

